# Reading chest radiographs in the critically ill (Part II): Radiography of lung pathologies common in the ICU patient

**DOI:** 10.4103/1817-1737.53349

**Published:** 2009

**Authors:** Ali Nawaz Khan, Hamdan Al-Jahdali, Sarah AL-Ghanem, Alaa Gouda

**Affiliations:** *King Fahad Hospital, King Abdulaziz Medical City, Riyadh, Saudi Arabia*

**Keywords:** Chest x-ray, intensive care unit, cardiopulmonary disorders

## Abstract

This is part II of two series review of reading chest radiographs in the critically ill. Conventional chest radiography remains the cornerstone of day to day management of the critically ill occasionally supplemented by computed tomography or ultrasound for specific indications. In this second review we discuss radiographic findings of cardiopulmonary disorders common in the intensive care patient and suggest guidelines for interpretation based not only on imaging but also on the pathophysiology and clinical grounds.

Interpreting chest radiographs in the critically ill patients in intensive care units (ICU) poses a challenge not only for the intensive care physicians but also for the radiologist. These challenges arise because of several factors:[1] ICU patients are prone to several cardiopulmonary disorders which when superimposed on the underlying pathology that prompted admission creates a complex radiological appearance, which may be difficult to interpret on imaging findings alone.[2] The standard postero-anterior (PA) radiograph is replaced by the suboptimal AP radiograph in the ICU patient.[3] Instrumentation, mechanical ventilation, equipment for monitoring of cardiac and other vital signs, feeding tubes, etc., distract from other findings on the ICU chest radiograph.[4] Radiologists/Intensive care physicians are under pressure for rapid interpretation of chest x-rays when treating critically ill patients, often with inadequate clinical information partly due to the fact that things can change rapidly in the critically ill.[5] Radiological interpretation is hampered by the bewildering array of line placements in the ICU patient, where incorrect placement is not uncommon, which may not be obvious to the observer without clinical input.[6] Air space shadowing in the ICU patient may have identical appearances in a variety of cardiopulmonary pathologies. Although the imaging modality of choice in the ICU patient remains that of chest radiography, computed tomography is often performed as computerized tomography pulmonary artery (CTPA) with suspected pulmonary embolism. Ultrasound is used to confirm pleural and pericardial effusions and when pleural intervention is planned.

The aim of this paper is[1] to discuss the radiographic findings of cardiopulmonary disorders common in the ICU patient and suggest guidelines for interpretation based not only on the chest radiograph but also on the pathophysiology and clinical grounds;[2] to describe the normal position of monitoring devices and correct placement of other lines, and prompt recognition when they are misplaced or when other complications occur. We discuss correct placement of, as well as common complications due to, monitoring lines.

This is a two-part series:[1] Part I: Normal chest radiographic appearances in the ICU patient, correct and incorrect placement of various intrathoracic tubes and lines and complications from instrumentation. Part II: Radiography of lung pathologies common in the ICU patient.

## Pulmonary Edema

Pulmonary edema is secondary to accumulation of fluid in the lung interstitium or alveolar space. Pulmonary edema is frequently seen and is a common cause of oxygen desaturation in the ICU patient. Several mechanisms are implicated in the genesis of pulmonary edema, including increased hydrostatic gradient, increased oncotic pressure or increased capillary permeability. One or a combination of these mechanisms may be involved. Pulmonary edema is broadly subdivided into cardiac and noncardiac. Cardiac edema is usually secondary to poor cardiac function, whilst noncardiogenic pulmonary edema can result from volume overload, diminished oncotic pressure or from endothelial injury as in the patient with adult respiratory distress syndrome (ARDS).

**Figure 1:**
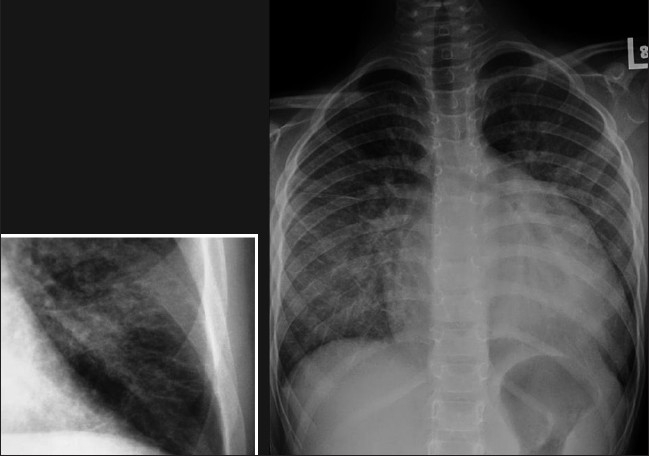
Frontal chest radiograph (right) showing features of interstitial pulmonary edema. Radiographic signs (shown in the figure) that suggest interstitial pulmonary edema include loss of definition of large pulmonary vessels, the appearances of septal lines, interlobar septal thickening and diffuse reticular pattern associated with cardiomegaly. Both Kerley's A and Kerley's B lines are seen. The magnified view of the left costophrenic angle is from another patient, depicting Kerley's B lines (left)

Interstitial edema results from fluid collection in the lung interstitial space and usually develops when the pulmonary venous pressure rises to 25-30mm Hg. Interstitial pulmonary edema is one condition which may be seen on a chest radiograph before symptoms develop. Radiographic signs that suggest interstitial pulmonary edema include loss of definition of large pulmonary vessels, the appearances of septal lines, interlobar septal thickening, diffuse reticular pattern resembling interstitial fibrosis and peribronchial cuffing seen as bronchial wall thickening as a result of fluid retention in the lung interstitium. Septal lines represent fluid in the deep septae and lymphatics and appear as[1] Kerley's A lines, which range from 5 to 10cm in length and extend from the hilum of the lung toward the periphery in a straight or slightly curved course; and[2] Kerley's B lines, approximately 2cm long, seen in the periphery of the lower lung, extending to the pleura [Figure 1].

**Figure 2:**
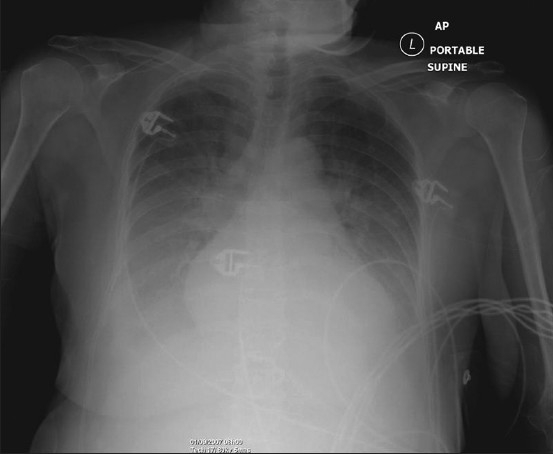
Frontal chest radiograph showing features of alveolar pulmonary edema. The findings include opacification of both lungs with increasing density towards the lung bases due to a combination of air space shadowing and pleural effusions, cardiomegaly, upper lobe blood diversion (unreliable on supine AP radiograph) and an air bronchogram in the right upper zone

**Figure 3:**
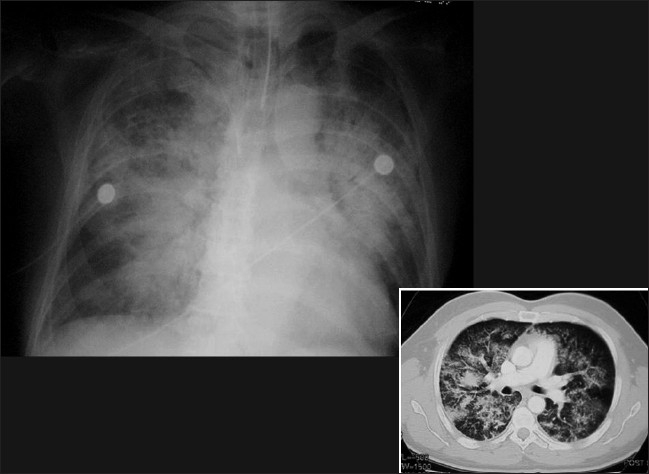
A frontal chest radiograph and axial CT show features of ‘batwing’ alveolar pulmonary edema. Chest radiographic findings include bilateral opacities that extend in a fan shape outward from the hilum in a batwing; pattern. With worsening alveolar edema, the lung opacification becomes increasingly homogenous

**Figure 4:**
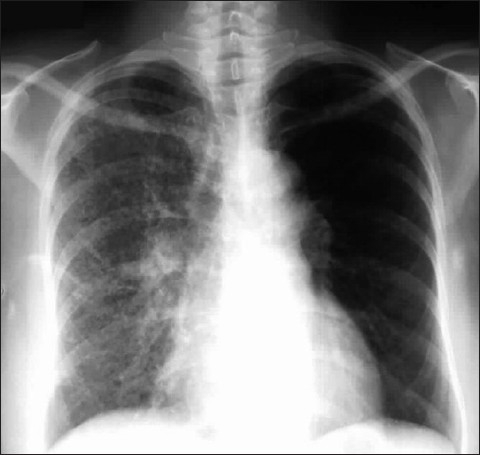
Supine portable chest radiograph showing extensive air space shadowing throughout the whole of the right lung and the left lung base due to alveolar pulmonary edema with associated pleural effusions secondary to heart failure. Note the air bronchograms in the right upper zone, sometimes seen with congestive heart failure

Alveolar pulmonary edema generally develops when the pulmonary venous pressure exceeds 30 mm Hg and is usually preceded by interstitial pulmonary edema [Figure 2]. Chest radiographic findings include bilateral opacities that extend in a fan shape outward from the hilum in a ‘batwing’ pattern [Figure 3]. With worsening alveolar edema, the lung opacification become increasingly homogenous. Normally the bronchi in the lung periphery are not seen because of air density within the bronchi and the surrounding lung parenchyma. However, along with fluid-filled alveoli from pulmonary edema or infection (pneumonia), the air-filled bronchi can be easily seen, an appearance known as ‘air bronchogram’ [Figure 4]. Air bronchograms associated with congestive heart failure are usually visible in the right upper lobe. In pulmonary edema due to heart failure, the heart size is often enlarged.

**Figure 5:**
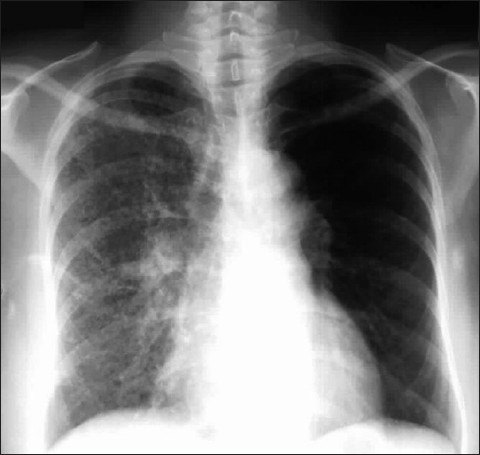
A frontal chest radiograph showing a unilateral edema

Diagnosis of pulmonary edema is not always straightforward, and atypical patterns can present diagnostic difficulties on radiographic findings alone. Atypical radiographic patterns of pulmonary edema include unilateral, lobar, miliary or lower-zones edema; and other asymmetric or unusual distribution patterns [Figure 5]. Miliary edema may precede full-blown lung edema. Lower-zones edema and lobar pulmonary edema generally occur in patients with chronic obstructive pulmonary disease and pulmonary emphysema.

Congestive cardiac failure causing cardiogenic pulmonary edema is usually the result of left ventricular failure, which is in turn due to poor cardiac output and increased pulmonary venous hydrostatic pressures. Generally it is a combination of a failing cardiac pump and fluid overload that tips patients into congestive heart failure. The chest radiograph is an important diagnostic tool in distinguishing fluid overload or congestive failure. This diagnosis of a left-sided cardiac failure due to an acute ischemic cardiac insult is suggested on the chest radiographs in 25% to 40% of patients before the onset of symptoms. Ideally the best technique in this setting is a standard PA chest radiograph because the accuracy of detecting cardiomegaly and redistribution of pulmonary blood flow on supine AP films is poor. Obtaining an erect PA radiograph may not be always possible in an ICU patient; and therefore as a compromise, semi-erect and decubitus films are recommended. Cardiomegaly, increased pulmonary vasculature, and pleural effusions are evident in the patient suffering from congestive heart failure.

A chest radiograph may differentiate between cardiac and noncardiac pulmonary edema. The radiographic features of cardiac edema include cardiomegaly, pleural effusions, upper lobe blood diversion, septal lines, peribronchial cuffing and basal edema. The only exception where the aforementioned changes have not had time to develop is acute myocardial infarction. There are a multitude of causes of noncardiogenic pulmonary edema, which include inhaled irritants causing a more mottled appearance and more peripheral distribution of lung parenchymal changes. Other causes of noncardiogenic pulmonary edema include near-drowning, altitude sickness, oxygen therapy, transfusion reactions, fat embolism, central nervous system disorder, ARDS or aspiration, renal disorder or/and drug reactions, to name just a few.[1–15].

## Adult Respiratory Distress Syndrome

Adult respiratory distress syndrome (ARDS) is a term applied to a syndrome where signs and symptoms of pulmonary edema occur in the absence of elevated pulmonary venous pressures. ARDS is associated with high mortality, as much as 50%, and is common in the ICU patients. ARDS results from a variety of causes, including sepsis or pulmonary infection, severe trauma, and aspiration of gastric contents.[17] The final pathway in ARDS is common to all causes, which is damage to the alveolar capillary endothelium, increased vascular permeability, and subsequent development of first, interstitial, and then, alveolar pulmonary edema. Patients with ARDS present with severe respiratory distress characterized by marked hypoxia that responds poorly even to administration of high concentrations of oxygen. The pulmonary capillary wedge pressure is generally normal, but there is decreased surfactant production, which leads to poor lung compliance and atelectasis that results in an intrapulmonary shunt with perfusion but no effective ventilation. Positive end-expiratory pressure can help to decrease atelectasis and shunting while improving oxygenation. The ultimate prognosis is variable: whilst some may recover fully, others progress to pulmonary fibrosis. There is some correlation between the duration and severity of ARDS and long-term complications. Prognosis is also dependent on age and preexisting COPD.

**Figure 6:**
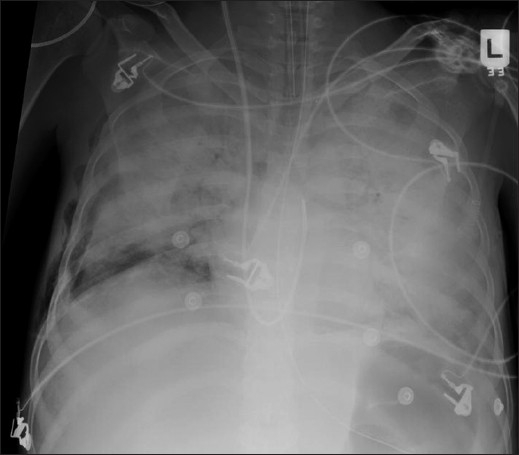
A patient's AP chest radiograph showing worsening of the air space shadowing with a further complication of a right-sided pneumothorax

**Figure 7:**
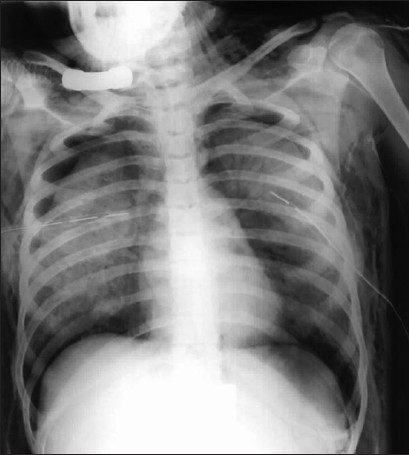
AP radiograph of the same patient with ARDS as in Figure 6 with further complication of bilateral pneumothoraces secondary to pleural drain placement

**Figure 8:**
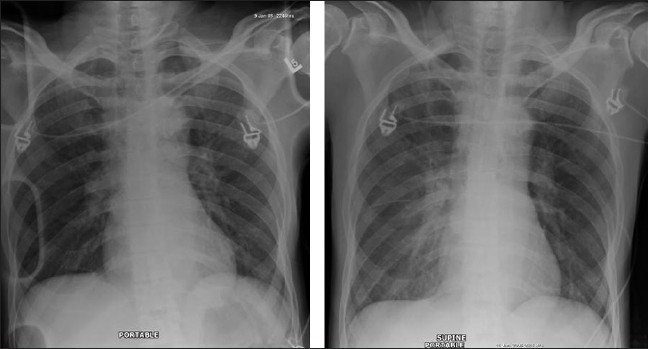
Figures 8, 9 and 10 show a series of chest x-rays and CT scans over a period of 18 hours of a patient following blunt thoracic trauma. The initial chest x-ray [Figure 8] appears normal

**Figure 9:**
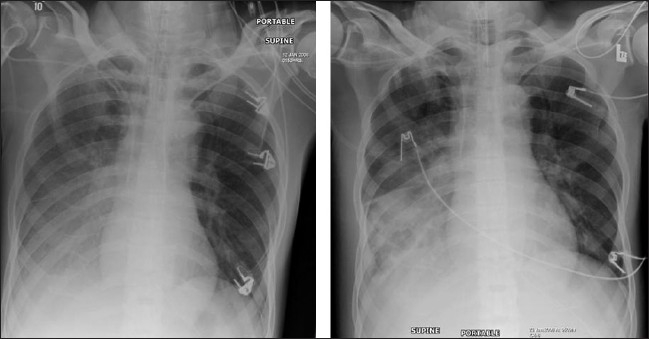
Same patient as in Figure 8; changes develop rapidly initially as mild opacification at the right lung base followed by lung parenchymal infiltrate associated with a small pleural effusion

**Figure 10:**
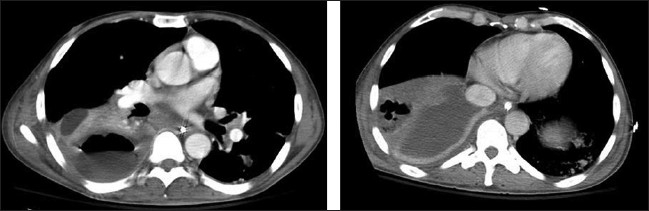
Same patient as in Figure 8; the opacity is in the peripheral lung, near the injured chest wall. The lesion rapidly progresses to cavitation as seen on the axial CT scans. The appearances are those of a lung contusion. Contusion however usually occurs earlier, is usually localized to the area affected by injury (e.g., unilateral and lower or upper zones) and improves over 48-72 hours. ARDS tends to be more generalized, is later in onset and slower to resolve

Differentiation between pulmonary edema of ARDS and congestive heart failure on the basis of radiographic signs alone can be challenging; moreover, the two conditions may coexist [Figures 6 and 7]. Although both entities may share the chest x-ray finding of bilateral air space opacification or ‘white out,’ ARDS is not usually associated with cardiomegaly or upper lobe blood diversion; however, upper lobe blood diversion is difficult to discern in the presence of air space opacification and on AP chest supine radiograph. Air space opacification in CHF can occur in the presence of a normal-sized heart. To make the issue more complicated, patients with ARDS could also have preexistent cardiomegaly or be fluid overloaded because of sepsis.[17–23] Lung contusion may be difficult to differentiate from ARDS. Contusion however usually occurs earlier, is usually localized to the area affected by injury (e.g., unilateral and lower or upper zones) and improves over 48-72 hours. ARDS tends to be more generalized, is later in onset and slower to resolve [Figures 8–10].

### Atelectasis

Atelectasis occurs when there is failure of the lung to expand (inflate) completely. This may be caused by any process which reduces alveolar ventilation, including blocked airway, e.g., obstruction from mucus plugging, a tumor, general anesthesia, pneumonia, splinting from pain following surgery.[28,32,33] Extensive alveolar hypoventilation may cause hypoxia as a result of an effective right-to-left shunt. It is a common abnormality seen on the ICU patient's chest radiograph. Atelectasis in ICU patients is seen most frequently in the left lower lobe. It is postulated that this is due to compression of the lower lobe bronchus by the heart, in the supine patient. A contributory factor may be relatively difficulty of blind suctioning of the left lower lobe. Usually atelectasis is more extensive than is suggested by the radiograph. Atelectasis can both be reversed and prevented with the use of hyperventilation and incentive spirometry, particularly in the postoperative patient.

Atelectasis may vary from a total lung collapse to subsegmental collapse to relatively normal-appearing lungs on the chest radiograph as an acute mucus plugging may cause only a small reduction in lung volume without visible abnormality. Notwithstanding radiographic appearances, the physiological effects may be significant. In a mucus plug syndrome, a sudden onset of hypoxia in the presence of a normal-looking radiograph can raise the suspicion of a pulmonary embolus and warrant an unnecessary CTPA. The radiographic features of atelectasis are summarized in Table 1.

**Table 1:** Radiographic features of atelectasis

Elevation of a hemidiaphragm
Displacement of a fissure
Crowding of the vasculature
Splaying of the vasculature seen in the non-affected lobe due to
compensatory emphysema
Mediastinal shift
Silhouetting

**Figure 11:**
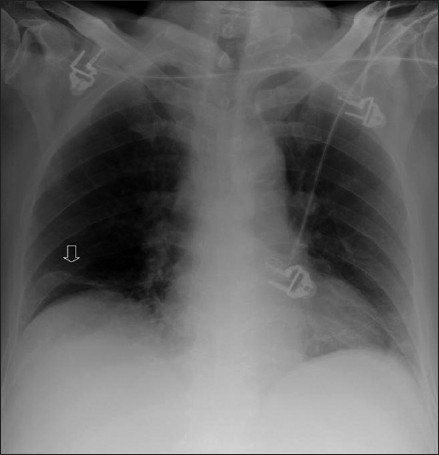
Plate atelectasis/discoid atelectasis (arrow) is common following thoraco-abdominal surgery and administration of a general anesthetic

Minimal basal subsegmental or discoid atelectasis appearing as linear streaks is common in the ICU patient and may not be physiologically significant [Figure 11]. Atelectasis may also mimic pulmonary consolidation, which may be difficult to distinguish from other causes of consolidation. To distinguish between atelectasis-related consolidation and consolidations related to other causes is important, and certain distinguishing features do exist. Atelectasis will often respond to increased ventilation while other causes of pulmonary consolidation will not. Other features that suggest atelectasis because of loss of volume in the involved lung include crowding of pulmonary vessels, displacement of interlobar fissures and elevation of the hemidiaphragm towards areas of atelectasis. Collapsed lung segments and lobes also follow well-recognized anatomical pathways, unlike other causes of consolidation.

The right upper lobe collapses into a triangular opacity, with the lesser fissure migrating toward the anterior, superior and medial portion of the chest, closing like a Chinese fan. On an AP chest radiograph, the most striking feature is a superior and medial displacement of the minor fissure. On the lateral radiograph, the major fissure moves anteriorly, while the superior movement of the minor fissure is also seen [Figurea 12 and 13].

**Figure 12:**
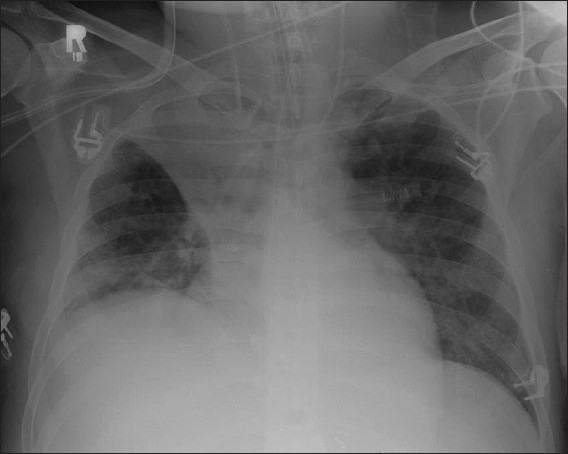
The right upper lobe collapses into a triangular opacity with the lesser fissure migrating toward the anterior, superior and medial portions of the chest, closing like a Chinese fan. On an AP chest radiograph, the most striking feature is a superior and medial displacement of the minor fissure. Note also the raised right hemidiaphragm. On the lateral radiograph (not shown), the major fissure moves anteriorly, while the superior movement of the minor fissure is also seen. This atelectasis was secondary to a mucusplug

**Figure 13:**
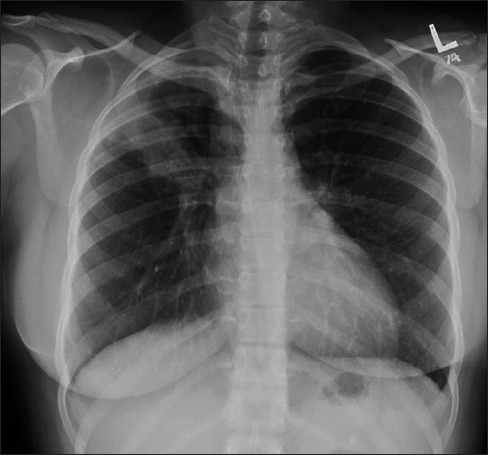
A frontal radiograph shows a segmental collapse of the right upper lobe. Note the elevation of the lesser fissure and the right hilum and a minor mediastinal shift to the right. This was an asthmatic patient, with a mucus plug

**Figure 14:**
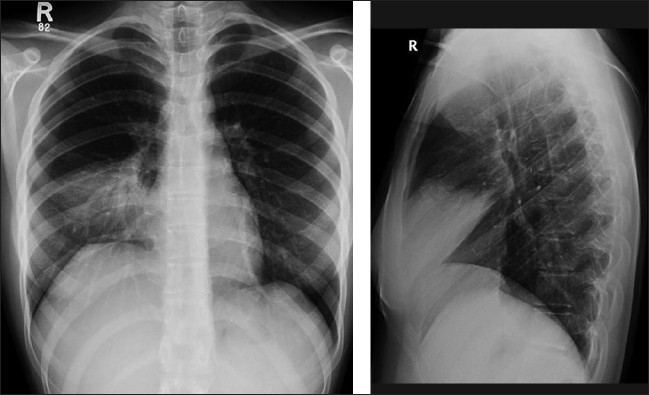
Right–middle-lobe atelectasis may cause minimal changes on an AP supine chest radiograph. Note the loss of definition of the right heart border. A collapsed right middle lobe is more clearly defined on lateral radiograph, which is not commonly available in the ICU patient. Attention to the fissures reveals that the horizontal and lower portions of the major fissures move towards each other resulting in a wedge of opacity pointing to the hilum. This is a middle-lobe consolidation mimicking middle-lobe atelectasis

Right–middle-lobe atelectasis may cause minimal changes on an AP supine chest radiograph. A constant feature is loss of definition of the right heart border. A collapsed right middle lobe is more clearly defined on lateral radiograph, which is not commonly available in the ICU patient. Attention to the fissures reveals that the horizontal and lower portions of the major fissures move towards each other resulting in a wedge of opacity pointing to the hilum. A middle-lobe atelectasis may mimic middle-lobe pneumonic consolidation [Figure 14].

**Figure 15:**
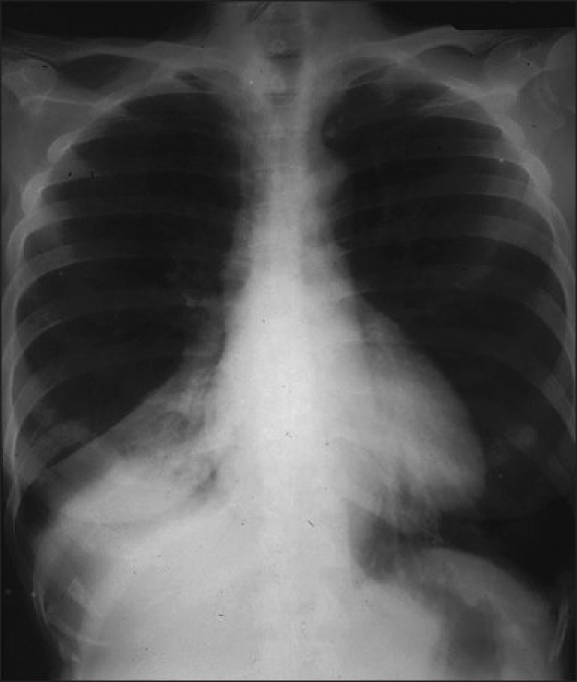
An AP chest radiograph showing atelectasis of the right lower lobe. Note that the collapsing lobe has moved centrally and inferiorly towards the lower dorsal spine, where it is seen as a triangular opacity partially silhouetting the right hemidiaphragm and associated with a subtle air bronchogram. The minor fissure shows inferior displacement. Right–lower-lobe atelectasis can be differentiated from right–middle-lobe atelectasis by the persistence of the right heart border as in this case

**Figure 16:**
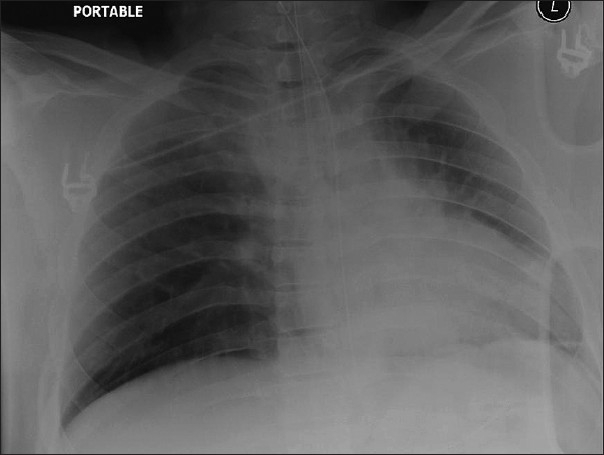
The left lower lobe collapses medially and posteriorly to lie behind the heart. It classically displays a triangular opacity, which may be visible through the cardiac shadow or may overlie it, giving the heart an unusually straight lateral border. Silhouetting of the left hemidiaphragm usually occurs, which may be associated with an air bronchogram. It is also easily missed, especially on an underpenetrated film, where no detail is seen behind the heart

Atelectasis of either the right or left lower lobe presents a similar appearance. In right–lower-lobe atelectasis the collapsing lobe moves centrally and inferiorly towards the lower dorsal spine, where it is seen as a triangular opacity. Silhouetting of the right hemidiaphragm and air bronchograms is a common sign of right–lower-lobe atelectasis. The minor fissure shows some inferior displacement. As the right lower lobe collapses, part of the greater fissure may become visible on the AP radiograph. A lateral radiograph, if obtained, may show inferior and posterior displacement of both the major and minor fissures. Right–lower-lobe atelectasis can be differentiated from right–middle-lobe atelectasis by the persistence of the right heart border [Figures 15 and 16].

**Figure 17:**
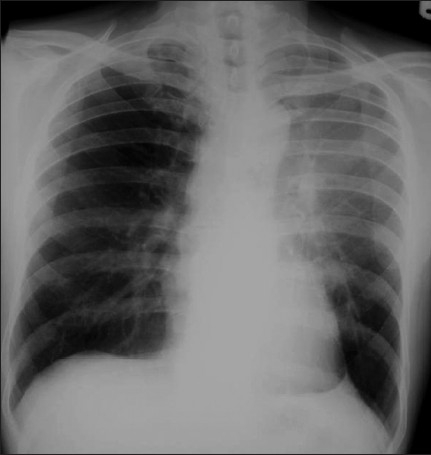
A frontal chest radiograph showing a left–upper-lobe atelectasis. The radiograph reveals hazy opacification of the left hilum, elevation of the left hilum, near-horizontal course of the left main bronchus, posterior leftward rotation of the heart and the Luftsichel or air crescent sign, the name given to the appearance of aerated lung abutting the arch of the aorta, between the mediastinum and the collapsed left upper lobe. An appearance on a lateral radiograph, if available, of the ICU patient may show retrosternal opacity and displacement of the greater fissure anteriorly

A left–upper-lobe atelectasis presents a different pattern compared to a right–upper-lobe atelectasis as the left lung lacks a minor fissure. When the left upper lobe collapses, the lobe predominantly moves anteriorly, with loss of the left upper cardiac border. There is compensatory emphysema of the left lower lobe, which expands and migrates to a location both superior and posterior to the left upper lobe. The left main bronchus also rotates to a nearly horizontal position. The AP chest radiograph reveals hazy opacification of the left hilum, elevation of the left hilum, near-horizontal course of the left main bronchus, posterior leftward rotation of the heart and the Luftsichel or air crescent sign, the name given to the appearance of aerated lung abutting the arch of the aorta, between the mediastinum and the collapsed left upper lobe [Figure 17]. An appearance on a lateral radiograph, if available, of the ICU patient may show retrosternal opacity and displacement of the greater fissure anteriorly.

The left lower lobe collapses medially and posteriorly to lie behind the heart. It classically displays a triangular opacity, which may be visible through the cardiac shadow or may overlie it, giving the heart an unusually straight lateral border. Silhouetting of the left hemidiaphragm usually occurs, which may be associated with an air bronchogram. It is also easily missed, especially on an underpenetrated film, where no detail is seen behind the heart.[18–36]

## Pneumonia in the ICU

**Figure 18:**
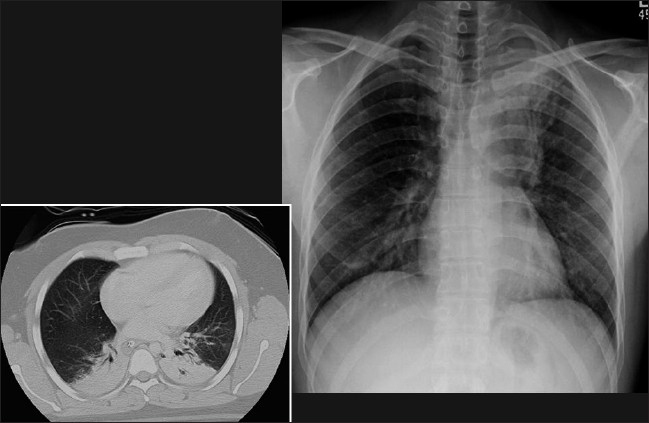
A chest radiograph (right) showing consolidation of left upper zone associated with an air bronchogram secondary to hospital-acquired pneumonia. The left image is an axial CT scan depicting an air bronchogram with bilateral pneumonic consolidation in another patient

**Figure 19:**
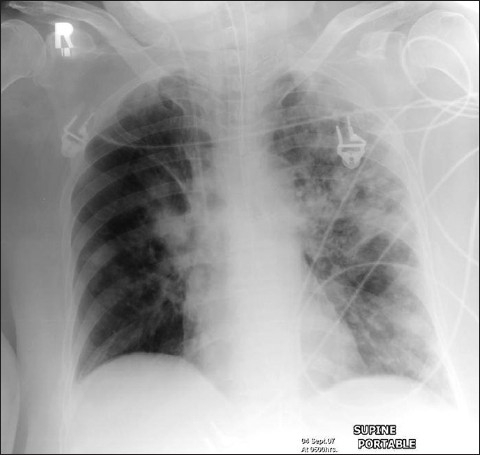
An AP supine radiograph on an intubated patient showing patchy consolidation in both lung fields, more prominent on the left due to hospital-acquired pneumonia

Hospital-based (nosocomial) pneumonias, which by definition occur 3 days after hospital admission, differ from community-acquired pneumonias in both causation and prognosis. Nosocomial pneumonia is the leading cause of death in the ICU patient.[40] The ICU patients are particularly susceptible to pneumonias as they may be immune compromised and several iatrogenic factors are at play, which increase this susceptibility. Iatrogenic factors that may predispose to pneumonias include endotracheal tubes; risk of aspiration; medications used to reduce gastric acid, which may promote bacterial growth in the stomach; and the use of antibiotics, which may selectively encourage the growth of some pathogenic bacteria. Unlike community-acquired pneumonias, which usually are caused by gram-positive species, nosocomial pneumonias are often polymicrobial and caused by gram-negative enteric pathogens. Clinical and laboratory findings such as fever, leucocytosis and sputum cultures may not be useful indicators and are often masked by severe underlying disease. The chest film must be correlated with clinical data in order to make the diagnosis of pneumonia in the ICU patient. Radiographically pneumonias can be difficult to differentiate from other causes of air space shadowing, including atelectasis and early ARDS. Usually pneumonia initially appears as patchy consolidation or ill-defined nodules [Figure 18]. Pneumonia is a bilateral multifocal disease and often involves gravity-dependent areas of the lung [Figure 19]. Atelectasis and lung edema have a similar distribution, making differentiation from pneumonia difficult. A symmetric pattern simulating pulmonary edema can occur with *E-coli* and pseudomonas pneumonias, which can rapidly involve the entire lungs. Patchy air space shadowing, ill-defined segmental consolidation or air bronchograms — either of these with associated pleural effusions supports the diagnosis of pneumonia. However, unlike community-acquired pneumonia, pleural effusions caused by gram-negative organisms are more likely to represent empyema and therefore require drainage. Other serious complications of pneumonias in the ICU patients include abscess formation and bronchopleural fistulas.[37–40]

## Aspiration

**Figure 20:**
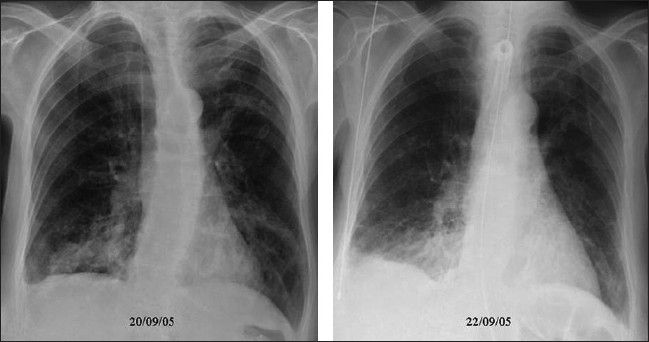
An AP chest radiograph of a patient with tracheostomy showing development of aspiration pneumonia at the right lung base

**Figure 21:**
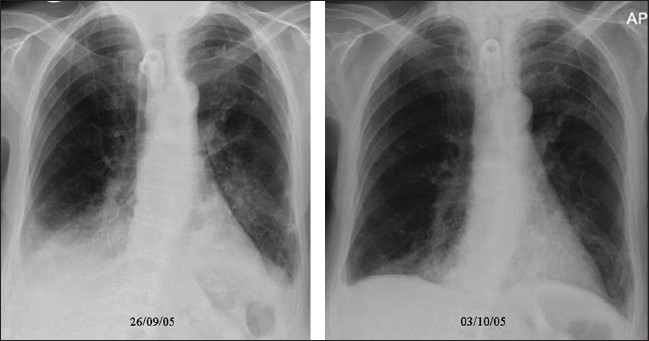
A series of AP radiographs on the same patient as in Figure 20 showing evolution of aspiration pneumonia at the right lung basewithin

The ICU patient is at a particular risk of aspiration pneumonitis, often as a result of a compromised airway. Impaired consciousness, placement of ET and NG tubes are amongst many contributory factors. The pulmonary response to aspiration depends on the type, pH and volume of aspirate. Aspiration of gastric contents provokes a chemical pneumonitis called Mendelson's syndrome. The lung responds to pH <2.5 with severe bronchospasm and the release of inflammatory mediators. Patients become symptomatic almost immediately following aspiration of gastric contents, with cough, dyspnea, wheezing and diffuse crackles. Fever and leucocytosis are the norm. The initial response is a chemical pulmonary edema. Secondary infection occurs in some cases. Patient response to aspiration varies from shock to resolution without sequelae. Patients that proceed to pneumonitis may reveal pulmonary consolidation within the first two days. The air space shadowing is bilateral, usually perihilar although asymmetric. Radiographically the consolidation usually begins to resolve by the third day [Figures 20 and 21]. In some, the consolidation may worsen with added complications of lung abscesses and pleural effusion.[41,42]

## Pulmonary Embolism

Morbidity and mortality from pulmonary embolism in the ICU remain high, and they continue to be underdiagnosed in the intensive care setting. Pulmonary embolism in the ICU setting may be completely silent, but it is a cause of sudden death. Symptoms are nonspecific and include dyspnea, tachypnea, hemoptysis, hypoxemia and pleuritic chest pain. Many risk factors exist, most important amongst them being a history of a previous embolic event. Other risk factors include immobilization, trauma, surgery, shock, obesity, pregnancy, polycythemia vera and antithrombin-III deficiency. The pathophysiology of pulmonary embolism consists of both hemodynamic and respiratory embarrassment. Hemodynamic consequences occur when more than half the cross-sectional area of the pulmonary vascular bed is occluded by the embolic episode, leading to acute pulmonary hypertension, hypoxemia, respiratory failure and right-sided heart failure. Pulmonary infarction ensues rarely in the absence of associated bronchial arterial compromise. Typically, infarctions tend to be hemorrhagic and occur in the lower lobes.

**Figure 22:**
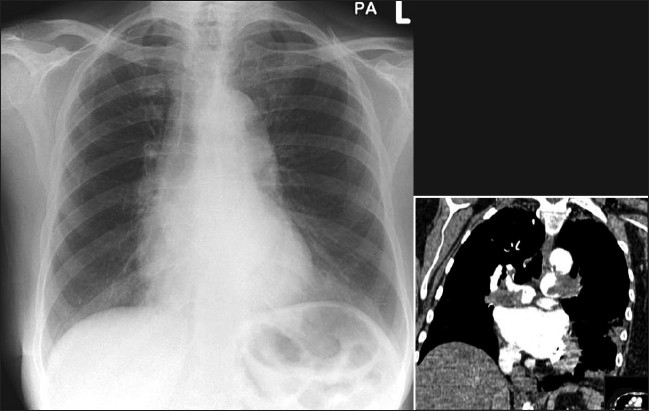
A frontal chest radiograph of a patient presenting with shortness of breath and hypoxemia, which shows no significant abnormality. However, CTPA (coronal reconstruction) shows extensive pulmonary embolism

**Figure 23:**
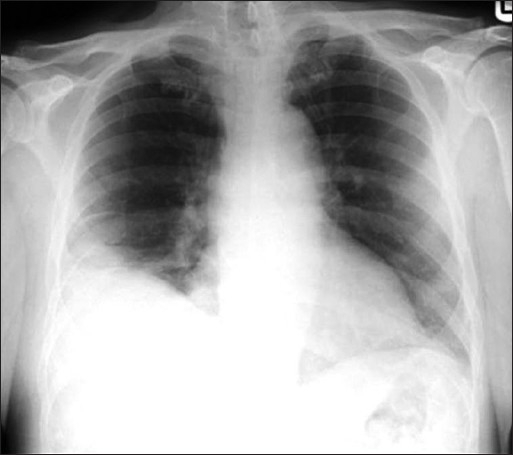
A relatively late sign of pulmonary infarction is a rounded pleural based consolidation that is rounded centrally and is called a Hamptom's Hump. A Hamptom,s Hump can be differentiated from a pneumonic consolidation as the former lacks an air bronchogram. Note also a small right costophrenic effusion tracking up into the lesser fissure

**Figure 24:**
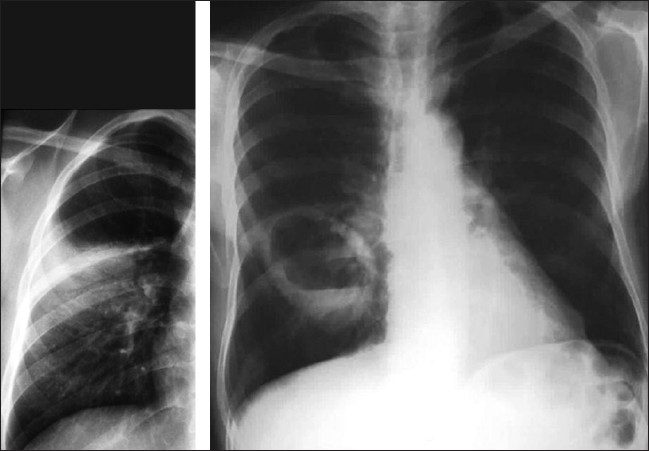
A pleural-based segmental opacity due to infarction (left), subsequently converting into a thick-walled cavity (right)

The chest radiograph has poor sensitivity in establishing the diagnosis of pulmonary embolism, and the role of a chest radiograph is in ruling out other pathologies that may have a clinical presentation similar to that of pulmonary embolism. The chest x-ray also serves a useful purpose when interpreting ventilation-perfusion scans. Studies have shown that the vast majority of patients with pulmonary embolism in retrospect do have abnormalities on the chest x-ray findings, but these findings are too nonspecific to be of clinical value [Figures 22–24]. In the absence of pulmonary infarction, only a few signs of pulmonary emboli are seen on a chest radiograph, which include discoid atelectasis, elevation of the hemidiaphragm, enlargement of the main pulmonary artery into what has been described as the shape of a ‘sausage’ or a ‘knuckle’ (Palla's sign), and pulmonary oligemia beyond the point of occlusion (Westermark's sign). A constellation of radiographic signs may be seen when pulmonary infarction complicates pulmonary embolism. Multifocal consolidation may follow with established pulmonary infarctions within 12 to 24 hours following the embolic episode. A relatively late sign of pulmonary infarction is a rounded pleural-based consolidation that is rounded centrally and is called a Hamptom's Hump. A Hamptom's Hump can be differentiated from a pneumonic consolidation as the former lacks an air bronchogram. Ipsilateral or bilateral pulmonary effusions although nonspecific are associated with approximately 50% of pulmonary emboli, although these are certainly nonspecific findings. Infarcts often are confused with, or are indistinguishable from, atelectasis or pneumonias on chest radiographs.

However, despite the low sensitivity of chest radiography in the diagnosis of pulmonary embolism, it remains an important first step in the diagnosis of pulmonary embolism, primarily to exclude other causes of hypoxemia and to aid in the interpretation of the ventilation/perfusion scan.[43–50]

## Conclusion

To summarize, radiography of lung pathologies common in the ICU patient is discussed, including pulmonary edema, ARDS, atelectasis, pulmonary embolism, aspiration and ICU-acquired pneumonia in terms of pathogenesis and radiographic recognition of the abnormalities. Differential diagnosis of the radiographic signs encountered is discussed. Reference is made where other imaging such as CT or ultrasound is indicated. A summary of radiographic recognition of atelectasis is presented.
